# Differential effects of stress-related and stress-unrelated humor in remitted depression

**DOI:** 10.1038/s41598-022-11515-y

**Published:** 2022-05-13

**Authors:** Anna Braniecka, Iwona Wołkowicz, Anna Orylska, Anna Z. Antosik-Wójcińska, Agnieszka Chrzczonowicz-Stępień, Ewelina Bolek

**Affiliations:** 1 Institute of Psychology, SWPS University of Social Sciences and Humanities, Chodakowska 19/31, 03-815 Warsaw, Poland; 2 Department of Psychiatry, Medical University of Warsaw, Warsaw, Poland

**Keywords:** Health care, Medical research, Risk factors

## Abstract

Enhancing emotion regulation among previously depressed people is crucial for improving their resilience and reducing relapse. Therefore, emphasis is placed on determining effective regulation strategies, particularly those that, besides down-regulating negative emotions, also up-regulate positive emotions. One promising strategy, with great potential in both these respects, is humor. It is unclear, however, what type of humor is most adaptive in remitted depression. This study compared two distinct humor-based strategies: *stress-related humor* and *stress-unrelated humor*. Outpatients with remitted depression (*N* = 94) participated in a randomized experiment evoking personal stress and the subsequent application of *stress-related humor*, *stress-unrelated humor*, or a non-humorous regulation. They repeatedly reported positive and negative emotions (at four time points) and experienced distress (at three time points). There were also assessments of selective attention, subsequent performance, effort, and intrusive thoughts. Unlike non-humorous regulation, humor-based strategies had adaptive consequences, both immediately and after a delay; however, *stress-unrelated humor* was most beneficial and was the only effective strategy when attention deficits were present. Humor, especially if unrelated to stressors, might broaden the repertoire of powerful emotion regulation strategies in remitted depression. Humorous focusing on distress can be detrimental for patients with attention impairment.

Clinical trial registration: The study was registered under the number ISRCTN86314628 (20/09/2021).

## Introduction

Distressing events are part of everyday life, and most people recover from them quite quickly. However, individuals vulnerable to depression face more challenges in this regard; they tend to employ maladaptive strategies for regulating emotions that sustain or escalate their stress and negative moods; this may ultimately lead to full-syndrome depression^1,2^. There is compelling evidence that dysfunctional emotion regulation is particularly pronounced in formerly depressed people, which makes their vulnerability to depression extremely acute^3^. Therefore, both clinicians and researchers have emphasized the need to determine which emotion regulation strategies are most adaptive and worth developing in depressive disorders^4–6^. Nonetheless, despite growing research efforts in this area, the findings are inconclusive. This appears to be due to a predominant focus on investigating strategies aimed at reducing negative emotions and related symptomatology without addressing positive emotions and well-being. The positive affect system is increasingly recognized as a significant treatment target for people with remitted depressive disorders, as it plays an essential role in building their resilience and buffering against future depressions^5,7^. Accordingly, it has been demonstrated that emotion regulation strategies that primarily focus on positive affect can be effective at both improving positive aspects of mental health and preventing and mitigating depressive symptoms^8^. Moreover, positivity-focused strategies of regulating emotional distress were found to predict the reduction of subclinical depression above and beyond the effect of traditional, negativity-oriented strategies^9^.

The present study aims to examine one promising positivity-oriented emotion regulation strategy, namely humor. The idea that humor has strong mood-enhancing benefits is widely accepted^10^, which is reflected in its identification as one of the 24 character strengths in the Classification of Character Strengths and Virtues (VIA)^11^. The research confirms that using humor in distressing contexts is associated with higher self-confidence, more frequent social interactions, and greater satisfaction with them^12^. Moreover, coping humor was proven to reduce negative affect, tension, and psychophysiological reactivity in both high and low trait-humor individuals^13^, which suggests that it can be an effective strategy even for people who do not typically use humor. Accordingly, there is empirical evidence that patients with major depression, despite their reported difficulties in using humor as a coping strategy, did not differ from healthy controls with respect to humor type preferences and the degree to which humorous material is rated as being funny^14^.

Indeed, there are some reports that the implementation of humor training among individuals with subclinical depression can improve can be effective, leading to improvements in positive (optimism, positive emotions, and self-efficacy) and negative (depressive symptoms, anxiety, and perceived stress) aspects of their psychological functioning^15,16^. Furthermore, a pilot study on the implementation of humor training in a group of patients diagnosed with depression provided preliminary evidence for the effectiveness of a group program designed to enhance humor abilities in a clinical setting^17^. Specifically, after 8 weeks of humor training, patients reported a short-term mood improvement and they were more capable of using coping humor. In addition, the acquired humor skills, along with the resulting positive emotions, were evidenced to help patients maintain motivation throughout the training period^17^.

Similarly encouraging findings have provided emotion regulation studies. For instance, generating funny comments for negative pictures was repeatedly found to both down-regulate negative emotions and up-regulate positive emotions^18–20^. There is also experimental evidence that being exposed to humorous material attenuates negative feelings to a greater extent compared to equally positive non-humorous stimuli^21^. In addition, a research on short humorous interventions and perception of stressful events demonstrated that humor is a powerful tool to attenuate both psychological stress response and salivary cortisol levels^22^. Only one study has investigated the impact of humorous emotion regulation strategies in remitted depression; it demonstrated that the use of humor by previously depressed people to comment on a series of distressing scenes could alleviate their negative emotions, increase positive emotions, and enhance distance from adversity^23^. Additionally, humor-based regulation was found to be more effective than spontaneous regulation, but contrary to initial expectations, humor did not prevail over positive reappraisal. Nonetheless, this result might be because participants could use any type of humor, although only some of them were effective emotion regulation tools for remitted depressed patients.

### Heterogeneity of humor in stressful contexts

Although humor has been traditionally understood in a unipolar and unidimensional manner, there is compelling evidence that not all uses of humor fit into this concept^24,25^. In fact, humor is a heterogeneous phenomenon which takes many different forms, each with a distinct set of emotional consequences^10,26^. Most studies in this area are predicated on the concept of four distinct styles of humor^27^; two of them are regarded as generally positive (i.e., affiliative and self-enhancing humor), and the other two are negative (i.e., aggressive and self-defeating humor). Following this concept, a functional magnetic resonance imaging (fMRI) study provided evidence of distinct neural correlates involved in processing different humor styles^28^. The majority of correlational studies to date suggest that positive styles of humor are beneficial for emotional health, while negative humor styles, particularly self-defeating humor, are associated with increased psychopathology^29^ and mediate between cognitive distortions and depressive symptoms^30^. Similarly, in their emotion regulation research, Samson and Gross^19^ demonstrated that producing positive (benevolent) humor to reinterpret adversity was more effective than producing negative (hostile) humor.

Nonetheless, research has increasingly emphasized that the relationship between the use of humor and its emotional effects cannot be fully explained by the emotional tone of the humor (positive vs. negative). For example, the experimental study of humor styles^31^ provided evidence that while engaging in self-enhancing humor did indeed result in lower anxiety, self-defeating humor did not have any negative effects. Moreover, an interview study with high scorers in self-defeating humor indicated that using humor at the expense of oneself was more strongly related to positive, rather than negative, emotions^32^. Accordingly, in the synthesis of the literature on humor-based emotion regulation, Samson and Gross^26^ contend that positive humor can be dysfunctional when used to deny problems or shield oneself from difficult feelings, while negative humor may sometimes serve adaptive functions, especially in critical life circumstances. Considering that vulnerability to depression is typically “triggered” in the face of a personally stressful experience^33^, we examined two basic kinds of humor, distinguished earlier by Strick et al.^21^ based on their relationship to the stressor involved.

### Stress-related humor and stress-unrelated humor

When a person prepares to use humor in distress, they first decide between one of two options: They either make fun of the stressor or find something else to joke about. The first option, which we have called *stress-related humor*, is based on focusing attention on an emotion-eliciting situation to transform its negative meaning into a humorous one. Accordingly, the main mechanism of *stress-related humor* is reappraisal, which is widely known as a powerful tool for improving emotions, self-esteem and psychological adjustment^34,35^. Humorous reappraisal has been investigated less than its serious form; however, the existing studies suggest that it can be based on de-emphasizing or positively reinterpreting adversity^36^, and it is generally more effective for regulating emotions than non-humorous reappraisal^20^. Interestingly, there is some evidence that individuals with more acute depressive symptoms report the mood-improving potential of humor, in the form of funny memes, to be greater when humor is related to depression and less effective when it is related to other topics^37^.

The second option for using humor, which we have described as *stress-unrelated humor*, relies on disengaging attention from the emotion-eliciting stimuli and transferring it to other humorous material. Thus, its main mechanism is distraction, commonly used as a seamless means to alleviate negative feelings, even under highly stressful circumstances^34^. The effectiveness of distraction results from preventing mood-congruent processing, which involves loading working memory with any mood-incongruent material^38^. Accordingly, the humorous form of distraction, with its very high cognitive demands, was shown to attenuate negative emotions to a greater extent compared to other forms^21^. However, it is worth noting that because the source of negative feelings was left intact, the emotional relief resulting from *stress-unrelated humor* may be ephemeral or involve maladaptive consequences related to encouraging emotional avoidance, deemed dysfunctional for depressive disorders^39^.

### The present study

The current study aimed to investigate two humor-based emotion regulation strategies, namely *stress-related humor* and *stress-unrelated hum*or, and compare their effectiveness with non-humorous regulation in improving negative emotions, positive emotions, levels of distress and intrusive thoughts in remitted depressed people. The three different emotion regulation conditions varied in a between-subjects design. There were four consecutive assessments (T1–T4) of the dependent variables: before and after stress induction (T1, T2), directly after an emotion regulation manipulation (T3), and after a delay (T4).

In line with the previous evidence of the beneficial impact of humor-based emotion regulation on remitted depression^23^, we expected that both *stress-related humor* and *stress-unrelated humor* would be effective in relation to all outcomes examined immediately and after a brief delay. We also presumed that, as demonstrated in previous clinical and non-clinical studies^23,38^, humor could impose some costs, such as requiring immense effort and compromising subsequent performance. Moreover, we expected that *stress-related humor* would be more effective than *stress-unrelated humo*r, as it facilitates confrontation with the stressor and shifts its threatening meaning through the powerful mechanism of humorous reappraisal. *Stress-related humor* could be particularly adaptive for individuals prone to depression in view the detrimental impact of their well-documented deficits in approach-related motivation^41^.

The study also aimed to investigate the importance of selective attention deficits which often persist in remitted depression, impairing emotion regulation ability^42–45^, for the effectiveness of humor-based regulation. Producing humor is cognitively demanding, especially in distressing situations, when it requires the simultaneous processing of both humorous and stressful stimuli. In this context, selective attention is pivotal, as it allows one to ignore or inhibit irrelevant (e.g., stressful) information and attend to relevant (e.g., humorous) information^46^. If so, participants with deficient selective attention would have difficulties shifting between these two sets of stimuli, which could result in an impaired application of humor and foster the relative effectiveness of non-humorous regulation.

## Results

### Statistical analyses

Verification of the main hypotheses was performed using repeated analyses of variance (ANOVA) measures, with positive emotions, negative emotions, and experienced distress as within-subject effects in consecutive measurements (time) and condition as the between-subject fixed factor. Effort, intrusive thoughts, and performance were analyzed using one-way ANOVA. To verify which emotion regulation strategy was most effective in improving negative emotions, positive emotions, and subjective distress, the difference scores between T2 and T3 (short-term effect) and between T2 and T4 (longer-term effect) were computed using one-way ANOVA. Selective attention was analyzed as the potential moderator of changes in dependent variables using repeated measures ANOVA, with dependent variables as within-subject effects and the condition and moderator as between-subject fixed factors. The data that support the findings are openly available in *Mendeley Data* at http://dx.doi.org/10.17632/9rc79r8nyw.1.

### Manipulation check

On average, participants complied with instructions well. Regarding the manipulation of emotion regulation, participants in the control (non-humorous) condition responded “sort of” (*n* = 15; 55.6%) or “yes” (*n* = 12; 44.4%) when asked about the rationality of the scenario. All participants in the *stress-unrelated humor* condition (*n* = 32; 100.0%) and all but one participant in the *stress-related humor* condition (*n* = 34; 97.1%) responded “no,” *λ*(4) = 86.61, *p* < 0.001. Regarding the funniness of the scenario, all participants in the control condition responded “no” (*n* = 27; 100.0%). In the *stress-unrelated humor* condition, 20 participants (62.5%) answered “sort of” and 12 responded “yes” (37.5%). In the *stress-related humor* condition, 20 participants (57.1%) responded “sort of,” 14 answered “yes’ (40.0%), and one chose “no” (2.9%), *λ*(4) = 105.51, *p* < 0.001. Participants who reported the use of strategies inconsistently with the condition were excluded from analysis (n = 11; 9.0%). There was no significant difference between the *stress-related humor* (*M* = 3.20, *SD* = 1.28) and *stress-unrelated humor* (*M* = 3.22, *SD* = 1.10) conditions in terms of the level of funniness of the scenarios, *t*(65) = −0.06, *p* > 0.05. The stress induction was also effective because negative emotions significantly increased between T1 and T2, *t*(93) = −10.16, *p* < 0.001, *d* = 1.01, while positive emotions diminished, *t*(93) = 8.74, *p* < 0.001, *d* = 0.98.

### Effects of emotion regulation strategies on emotions and experienced distress

**Figure 1:**
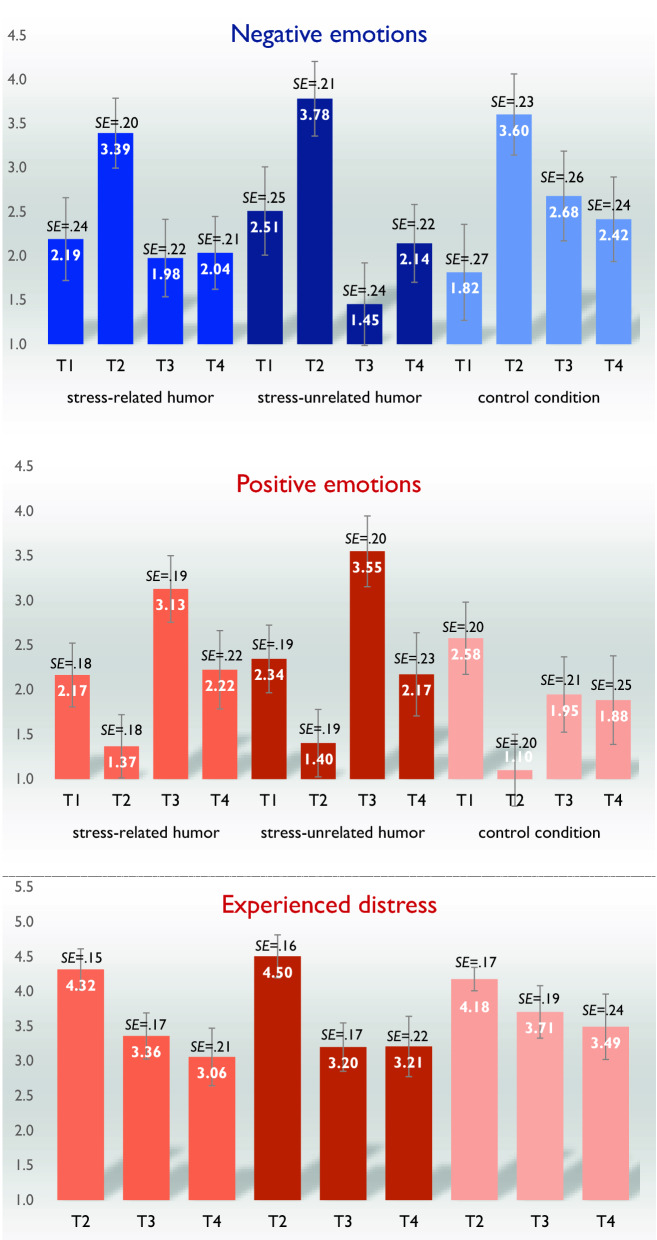
Mean values with standard errors (*SE*) of negative emotions, positive emotions, and experienced distress at four time points (T1*–*T4) in three experimental conditions (emotion regulation strategies), with 95% confidence intervals.

The results for negative emotions (Fig. 1) were significant in terms of time, *F*(3,261) = 2.72, *p* < 0.05, *η*^2^ = 0.03; however, there was also significant interaction between condition and time, *F*(6,261) = 4.75, *p* < 0.001, *η*^2^ = 0.10. In all conditions, negative emotions were significantly higher in T2 than in T1. In the *stress-unrelated humor* condition, negative emotions were significantly lower in T3 than in T1, *t*(31) = −4.38, *p* < 0.001, *d* = −0.79, and T2, *t*(31) = −9.47, *p* < 0.001, *d* = −1.70. In the *stress-related humor* condition, negative emotions were significantly lower in T3 than in T2, *t*(34) = −6.14, *p* < 0.001, *d* = −1.05, but there was no significant difference between T1 and T3, *t*(34) = −0.95, *p* > 0.05. In the control condition, there was no significant difference between negative emotions in T2 and T3, *t*(26) = 0.96, *p* > 0.05; however, they were lower in T4 than in T2, *t*(26) = −4.31, *p* < 0.001, *d* = −0.85. In the *stress-related humor* condition, negative emotions in T4 were close to the levels in T3, *t*(34) = 0.25, *p* > 0.05. In the *stress-unrelated humor* condition, negative emotions were lower in T4 than in T2, *t*(31) = −6.48, *p* < 0.001, *d* = −1.16, but they did not differ significantly from T1, *t*(31) = −1.27, *p* > 0.05.

The results for positive emotions (Fig. 1) revealed no significant time effect, *F*(2,69; 237.11) = 1.14, *p* > 0.05; however, there was significant interaction between condition and time effect, *F*(5,39; 237.11) = 6.47, *p* < 0.001, *η*^2^ = 0.13. In all conditions, positive emotions were significantly lower in T2 than in T1. In the *stress-related humor* condition, positive emotions were higher in T3 than in T1, *t*(34) = 4.37, *p* < 0.001, *d* = 0.75, and T2, *t*(34) = 7.93, *p* < 0.001, *d* = 1.36. In the *stress-unrelated humor* condition, positive emotions were higher in T3 than in T1, *t*(31) = 5.15, *p* < 0.001, *d* = 0.93 and T2, *t*(31) = 9.10, *p* < 0.001, *d* = 1.64. In the control condition, positive emotions were higher in T3 than in T2, *t*(26) = 3.37, *p* < 0.01, *d* = 0.66, but not higher than in T1, *t*(26) = −1.91, *p* > 0.05. In the *stress-related humor* condition, positive emotions were significantly lower in T4 than in T3, *t*(34) = −4.54, *p* < 0.001, *d* = −0.78. In the *stress-unrelated humor* condition, positive emotions were significantly lower in T4 than in T3, *t*(31) = −6.50, *p* < 0.001, *d* = −1.17. In the control condition, they were close to T3, *t*(26) = −0.27, *p* > 0.05.

Regarding experienced distress, there was no main time effect, *F*(2,176) = 1.66,* p* > 0.05; however, there was significant interaction between condition and time effect, *F*(4,176) = 2.54, *p* < 0.05, *η*^2^ = 0.06 (Fig. 1). In the *stress-related humor* condition, the distress level was significantly higher in T2 than in T3, *t*(34) = 5.26, *p* < 0.001, *d* = 0.90, and T4, *t*(34) = 6.51, *p* < 0.001, *d* = 1.12. Also, in the *stress-unrelated humor* condition, the distress level was significantly higher in T2 than in T3, *t*(31) = 6.85, *p* < 0.001, *d* = 1.23, and T4, *t*(31) = 6.40, *p* < 0.001, *d* = 1.15. In the control condition, no significant differences emerged. Since, depressive symptoms and coping styles correlated with negative emotions, positive emotions, and distress in all measurements, the BDI II^47^ and CISS^48^ scores were entered as covariates.

### Effects of emotion regulation strategies on effort, intrusive thoughts and performance

There were no significant between-group differences in effort, *F*(2,88) = 0.66, *p* > 0.05, *M* = 3.20, *SD* = 1.42, nor in performance (number of correct answers in the knowledge test), *F*(2,94) = 0.82, *p* > 0.05, *M* = 4.54, *SD* = 1.43. However, the number of intrusive thoughts was lower in the *stress-unrelated humor* condition (*M* = 0.83, *SD* = 1.09) than in the *stress-related humor* condition (*M* = 2.59, *SD* = 2.89) and the control condition (*M* = 1.88, *SD* = 2.41); *F*(2,87) = 4.72, *p* < 0.05, *η*^2^ = 0.10.

### Comparisons of the effectiveness of emotion regulation strategies

**Figure 2:**
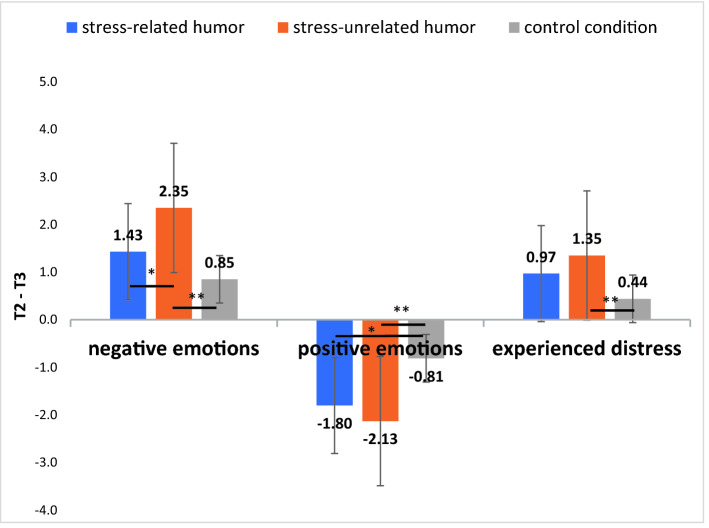
Differences between T2 (after recalling a personally stressful issue) and T3 (immediately after creating the scenario of events) in terms of negative emotions, positive emotions, and experienced distress in three experimental conditions (emotion regulation strategies). Arrows indicate significant differences.

There were three significant short-term differences for negative emotions, positive emotions, and experienced distress levels (Fig. 2). According to the Bonferroni pairwise comparison procedure the decrease in negative emotions was higher in the *stress-unrelated humor* condition than in the *stress-related humor* condition, *p* < 0.05, and the control condition, *p* < 0.01. The improvement in distress was significantly higher in the *stress-unrelated humor* condition than in the control condition, *p* < 0.01, but the differences between both humor conditions and between the *stress-related humor* and control conditions were insignificant, *p* > 0.05. The increase in positive emotions was significantly lower in the control condition than in both the *stress-related humor*,* p* < 0.05, and the *stress-unrelated humor* conditions, *p* < 0.01; there were no significant differences between the humor conditions, *p* > 0.05.

### Selective attention as a moderator between emotion regulation strategies and their outcomes

**Figure 3:**
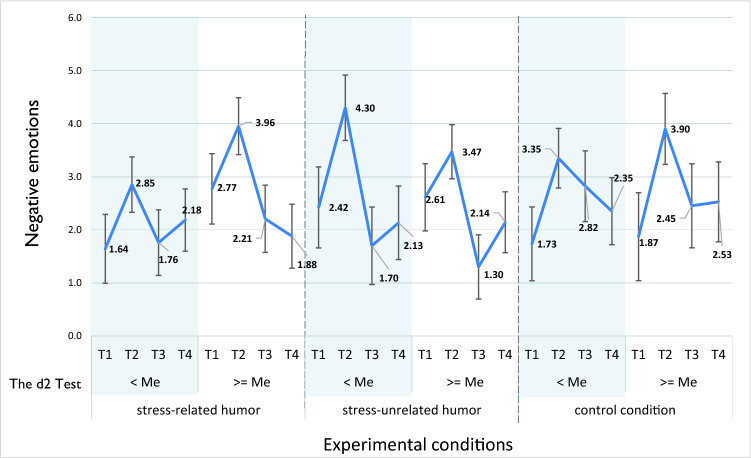
Estimated marginal mean values of negative emotions in four consecutive measurements (T1*–*T4) under three experimental conditions (emotion regulation strategies), depending on the d2 Test (*E*  total errors) score, with 95% confidence intervals (*Me*  median).

There were significant effects on the E score (total errors) of the d2 Test of Attention^47^, and it was examined as a possible moderator of changes in negative emotions, positive emotions, and distress under each condition. The participants were divided into two groups with the use of a median split (*Me* = 21.00). There were significant interactions between time effects and conditions in relation to negative emotions, positive emotions, and distress. In the analyses concerning negative emotions, there was also significant interaction between time effect, conditions, and selective attention. Figure 3 illustrates that the intensity of negative emotions in T3 and T4 returned to the preliminary level in most cases, with the exception of participants in the control condition and participants with d2 Test of Attention (E) scores lower than the median who were assigned to the *stress-related humor* condition. In these cases, there were no significant changes in the levels of negative emotions between T2, T3, and T4.

## Discussion

We aimed to investigate the consequences of two distinct kinds of humor, *stress-related humor* and *stress-unrelated humor*, for regulating distressing emotions in remitted depression. The results suggest that both types of humor improved negative emotions, positive emotions, experienced distress, and intrusive thoughts more effectively than the non-humorous regulation. They down-regulated negative emotions, both immediately and after delay, and up-regulated positive emotions to such an extent that they exceeded the initial level; however, their intensity decreased over time and, within 20 min, returned to baseline. The relatively rapid decline in positive emotions appears to conform with the phenomenon of hedonic adaptation, which is regarded as faster and more often complete to positive than to negative events^50^. Considering the strong social connotation of humor, it can be speculated that the effective means of inhibiting adaptation to humor-related positive emotions could involve their capitalization in an interpersonal context; for instance, this could involve sharing comical situations or humorous interpretations of distress with others.

Contrary to our expectations, humorous regulation did not demand greater effort than non-humorous regulation, and it did not jeopardize participants’ subsequent performance. This might result from the fact that our humor-generating procedure was substantially facilitated; the participants were provided with detailed guidelines and led step-by-step through the process of producing scenarios. Perhaps also for this reason, the manipulation of emotion regulation was highly effective: In both humor conditions, all participants reported that they applied a target strategy. The ability to successfully produce humor among previously depressed individuals was also revealed by Braniecka et al.^23^, which seems to encourage the promotion of humor as an emotion regulation strategy in this group. Indeed, although generating humor is generally difficult^26^, and patients with depression were shown to be less likely to use humor in the face of distress^14^, their susceptibility to humor-based regulation appears to be unaffected. In line with that, it was demonstrated that individuals’ ability to produce humor in distressing contexts is not compromised by depressive symptoms, negative mood states, or high perceptions of threat^51^. The result regarding unaffected subsequent performance is also consistent with previous non-clinical evidence that humor intervention during stress induction protects against psychological and physiological distress, leaving cognitive performance intact^22^.

The results concerning the differential effects of *stress-related humor* and *stress-unrelated humor* were inconsistent with our expectations that the former would prevail over the latter. Specifically, both types of humor had similarly beneficial impacts on positive emotions, and *stress-unrelated humor* was more effective in terms of negative emotions (even to a level lower than at the baseline), experienced distress, and intrusive thoughts. One potential explanation for these findings involves the powerful impact of the primary mechanism of *stress-unrelated humor*, namely humorous distraction. Humorous distraction is relatively simple and delivers quick emotional relief^52^, while remitted depressed people have an impaired ability to regulate emotions or execute more demanding strategies, such as reappraisal^3^. In addition, distraction entails the blockage of the emotion-generation process at the earliest possible stage before it accumulates force^53^, and prevents negative mood-congruent processing^21^. Therefore, it was discovered to reduce the critical process underlying emotional dysregulation in depression vulnerability: ruminating over negative emotion-eliciting stimuli^54^. Indeed, the high effectiveness of distraction in reducing negative affect is thought to be characteristic of currently and formerly depressive individuals; it was not observed to such an extent in never-depressed people^54^. In addition, the results regarding less beneficial consequences of *stress-related humor* seem to be consistent with neuroimaging evidence demonstrating abnormal default mode network subsystems connectivity, which is already observed in the first episode of depression and contributes to the pathophysiology of the maladaptive self-focus, involving repetitive and passive processing of stressful stimuli^55^.

In addition, the magnitude of the experimental effects among the three emotion regulation strategies was the largest for *stress-unrelated humor*. This was the case for negative emotions and experienced distress, as well as positive emotions. Strong effect of *stress-unrelated humor* on positive emotions may suggest that it has a greater exhilarating potential than *stress-related humor*. This might be due to its less aversive content, which does not include personally distressing information, making it easier for a person to respond with intense amusement or mirth. In view of the well-documented deficits in experiencing positive emotions in remitted depressed individuals, which in turn increase their vulnerability to further episodes, it seems that the substantive effect of *stress-unrelated humor* on enhancing positive experiences is of particular value. Altogether, considering that humorous forms of distraction involve higher cognitive demands compared to rational forms and also induce positive emotions, it appears that *stress-unrelated humor* may be indeed an effective, if only temporary, strategy of overcoming negative experiences by depression-prone individuals.

Notably, there is some evidence that distraction can be either adaptive or maladaptive, depending on how it is applied; it was advantageous if it was used with an accepting attitude, whereas it was detrimental if applied in the form of experiential avoidance^56^. As such, distraction associated with *stress-unrelated humor* appears to be based on acceptance rather than on avoidance. This would be consistent with the research on humor in the context of self-threat, demonstrating that humor-based regulation enables the appraisal of a negative event as simultaneously harmful and acceptable^40^.

The present study also examined the importance of selective attention impairment for the effective application of humor-based regulation. The results once again highlighted the prevalence of *stress-unrelated humor*, which was found to be the only effective strategy for people with selective attention deficits. More precisely, negative emotions returned to the preliminary level in most participants, with the exception of people who employed non-humorous strategy and those who applied *stress-related humor* and had attentional deficits. Among these participants, the intensity of negative emotions did not change after the use of the strategy; instead, it remained consistent through the end of the experiment. This finding can be explained by the fact that both *stress-related humor* and non-humorous strategy required focusing on a personally stressful issue during the generation of a scenario, which could be an insurmountable obstacle for people with impaired selective attention. They might have increased difficulties in controlling their depressive bias towards self-relevant negative stimuli, leading them to an intense preoccupation with their own distressing issue and ultimately preventing their recovery from negative affect. If so, it appears that emotion regulation tools that involve elaborating (humorously or rationally) upon a personally stressful issue should be applied in vulnerability to depression with great caution, as individuals with attentional deficits may have increased difficulties disengaging from stress-related negative information.

We also found that non-humorous strategy was ineffective for all participants, regardless of their attentional impairments. This seems to align with the generally dysfunctional spontaneous emotion regulation skills in remitted depression, especially in personally distressing contexts^5^. Notably, participants without selective attention deficits were found to benefit from *stress-related humor*, which suggests that this type of humor might buffer emotional oversensitivity to the self-relevant distress of depression-prone individuals.

Several limitations must be discussed. First, we measured the impact of emotion regulation in the short-term; however, *stress-related humor* and *stress-unrelated humor* can have different long-term effects. When stress relief is no longer a priority, shifting mental perspective on a distressing situation might become more relevant. In future research, it will be important to investigate these issues over a longer period. Second, because our study was an explorative one, it compared one application of each kind of humor, although different sub-types of distraction^56^ and humorous reappraisal^36^ can be distinguished. More studies are warranted in this area. Finally, the study did not include a non-humorous strategy that is unrelated to the stressor. Further research should incorporate this condition and examine to what extent the effectiveness of *stress-unrelated humor* in remitted depression can be attributed to distraction and other humor mechanisms.

This study is, to our knowledge, the first to compare different types of humor as emotion regulation strategies in remitted depression; its findings contribute to our understanding of adaptive regulating emotions among depression-prone individuals, underlining the importance of their cognitive limitations in this respect. In line with the previous research^23^, we provided evidence for the effectiveness of humor-based regulation, extending it to a self-relevant distressing context. Furthermore, within the boundaries of short-term consequences, we demonstrated that *stress-unrelated humor* can be a more beneficial emotion regulation strategy than *stress-related humor* and non-humorous strategy, and that this type of humor was the only effective strategy in reducing negative emotions when selective attention impairment was present.

## Methods

### Power analysis

The expected minimum effect size was assumed to have the value of partial eta squared: *η*^2^ = 0.05. The assumed statistical power had the value of 0.80, the level of significance was equal to the conventional level of 0.05. There were three independent groups to compare in terms of up to four consecutive measurements. The assumed correlation between repeated measurements was set to 0.5. Calculations done using G*Power 3.1.9.2 software led to the conclusion that the sample size should be at least 92 participants. Because the procedure involved a high risk that some participants would not complete the study, 122 participants were recruited.

### The sample

Participants were recruited from outpatient psychiatric clinics. The basic inclusion criterion was a diagnosis of remission after a depressive episode, made by a psychiatrist and confirmed via a Structured Clinical Interview (SCID I)^57^ administered by a clinical psychologist blind to the psychiatric diagnosis. An additional inclusion criterion was a BDI‐II^47^ score above a cut-off of 16, which is commonly used in studies on remitted depression because it ensures, at most, a mild intensity of depressive symptoms^58,59^. Exclusion criteria encompassed the previously applied set of health issues^23,60^ and involved the following: (a) history of manic or psychotic episodes, head injury, or neurological disorder and (b) current presence of eating disorders, anxiety disorders, intellectual disability, psychoactive substance use, pregnancy, or suicidal ideation.

The initially recruited sample consisted of 122 participants: 82 women and 40 men aged 18 to 65 (*M* = 42.30; *SD* = 12.49). Individuals for which acquired results were 1.5 times higher than the interquartile range from the 75th percentile or 1.5 times lower than the interquartile range from the 25th percentile were considered outliers, and cases for which the distance in a score distribution was higher than 3 times the interquartile range were considered extremes. We excluded 17 participants as outliers or extremes based on box plots drawn for each dependent variable, 11 participants for the sake of ineffectiveness of manipulation (individuals who reported the use of regulation strategy inconsistently with the condition), and one participant because of results below 3 *SD* from the mean for two dependent variables. The final sample consisted of 94 participants, namely 65 women and 29 men between the ages of 18 and 65 (*M* = 40.72; *SD* = 12.43). Information on medication is provided in Supplementary Material 1. The sample characteristics are presented in Table 1.

**Table 1:** Summary of sample characteristics.

	Frequency (%) (*n* = 94)	Mean (*SD*)	Statistics
**Demographic information**			
Age, years		40.72 (12.43)	
Gender			
Male	29 (30.9)		*χ*^*2*^_(1)_ = 14.00 *p* < 0.001
Female	65 (69.1)		
Employment			
Employed/in education	53 (56.4)		*χ*^*2*^_(4)_ = 103.66 *p* < 0.001
Not employed	28 (29.8)		
Time in education, years		13.11 (2.94)	
**Clinical information**			
BDI-II		12.20 (3.86)	
Main diagnosis			
First depressive episode	22 (23.4)		*χ*^*2*^_(1)_ = 8.96 *p* < 0.01
Recurrent depressive disorder	72 (76.6)		
Remission			
Full remission	53 (56.4)		*χ*^*2*^_(1)_ = 1.53 *p* = ns
Partial remission	41 (43.6)		
Lifetime number of episodes		3.98 (3.92)	
Age of first onset, years		30.78 (14.00)	
Number of admissions		1.24 (1.19)	
Comorbidities			
No	67 (71.3)		*χ*^*2*^_(1)_ = 17.02 *p* < 0.001
Yes	27 (28.7)		
Substance use—remission	8 (8.5)		
Anxiety disorders	19 (20.2)		
Personality disorders	1 (1.1)		

Participants were randomized into one of three groups; 35 participants were placed in the *stress-related humor* condition, 32 in the *stress-unrelated humor* condition, and 27 in the control condition (non-humorous regulation). There was no significant relationship between condition and gender, *χ*^2^(2) = 1.12, *p* > 0.05; medication, *χ*^2^(2) = 0.63, *p* > 0.05; or age, *F*(2,91) = 0.99, *p* > 0.05.

### Study design

**Figure 4:**
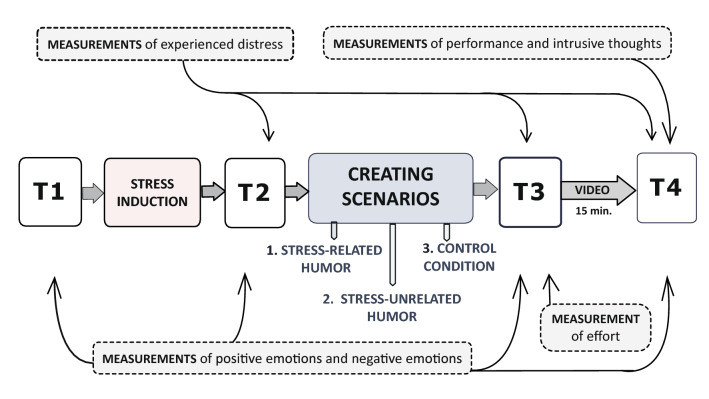
Flow diagram of the experimental part of the study.

Each person pre-qualified by a psychiatrist met with a clinical psychologist to complete the recruitment procedure, which was based on SCID I and BDI II fulfilment. The recruited individuals provided written consent after the study had been explained. They were informed that they were participating in a study on cognitive functioning and creativity in the face of personal distress. The introductory stage was followed by a neuropsychological assessment of selective attention, and the participants completed two additional questionnaires for a different research project. Then, within a week, they took part in a double-blind laboratory experiment; it consisted of stress induction (recalling any current personally stressful issue) and emotion regulation manipulation, which involved the application of one of the three strategies corresponding to the three experimental conditions: *stress-related humor, stress-unrelated humor,* and non-humorous regulation. There were also repeated measures of the dependent variables. Negative emotions and positive emotions were assessed four times: at baseline (T1), after stress induction (T2), after the emotion regulation manipulation (T3), and after a delay (T4). The experienced distress was measured in T2, T3 and T4. A single assessment was conducted to measure invested effort (T3), subsequent performance (T4) and intrusive thoughts (T4). The sequence of the experiment is illustrated in Fig. 4.

### Experimental procedure

Upon arrival at the laboratory, a participant was handed a printed packet which contained all experimental materials; they were then asked to read it carefully and follow the instructions without skipping any portions. The experimenter was present in the room at a distance of approximately 2 m. Repeated ratings were measured using single self-report scales. Initially, positive and negative emotions were rated on two scales of 0 (“not at all”) to 6 (“as strong as possible”) under the following question: “How strong are your positive/negative emotions at the moment?”. Then, to induce stress, the participant was asked to recall any of his/her own currently stressful issues to be addressed later in the study and to write a statement describing the selected issue. To facilitate this task, the participant was presented with an example: a short description of a stressful situation involving a person matched for age, gender, and history of depression. Next, to enhance their negative mood, we adapted the dysphoric mood induction procedure of Nolen-Hoeksema and Morrow^61^, in which the participant is asked to focus on the causes, meanings, and consequences of their negative issue and related feelings (for about 8 min) by reflecting on a series of thoughts (e.g., “Why do things turn out the way they do for me?”, “What might my current feelings mean?”). This was followed by again reporting their emotions and experienced distress (“How much stress are you feeling about your distressing issue?”), also on a scale from 0 to 6.

In the next step, the participants were assigned to experimental conditions and asked to produce a scenario in the form of a sequence of events. The randomisation sequence was generated by an independent statistician using a table of random numbers and was stratified by site using statistical software. A computer program generated one of three emotion regulation strategies for each participant according to the research allocation, so that experimenters and participants were not aware of individual assignments. In the *stress-related humor* condition, the scenario was supposed to be humorous and related to one’s own stress-inducing issue; in the *stress-unrelated humor* condition, it was supposed to be humorous and unrelated to one’s own stressful issue; and in the non-humorous condition, it was supposed to be rational and related to one’s own stressful issue. The experimental manipulation (see Supplementary Material 2) was derived from stress management techniques^62^, and it involved an exaggeration-based construction, conceptually overlapping with absurd humor. The humorous exaggeration has previously been used in experimental humor research, and it is considered an easy and universal comic device^63^.

In the *stress-related humor* condition, the participant started by writing down what they feared could happen because of the stressful issue and then answered a series of questions (“And then what?”) in an increasingly negative, exaggerated manner until the outcome became ridiculous. The person then moved on to an exaggerated positive continuation, responding in an increasingly optimistic way, and finished with an absurdly positive outcome. In the *stress-unrelated humor condition*, the procedure was the same except that the humorous scenario concerned the situation of an unknown fictional person. In that case, the participant received one of six descriptions of someone’s distress. To avoid the participant identifying with that person, he/she was of a different gender and worked in a gender-stereotyped profession. In the control condition, the scenario concerned the participant’s stressful issue; however, it was intended to be as realistic as possible. Therefore, they were instructed that the positive and negative parts of the scenario were supposed to be plausible. In each condition, the task was accompanied by an example from a pilot study.

As a manipulation check, the participants answered two questions, one about the subjective funniness of the scenario (“Does this scenario seem funny to you?”) and one about its rationality (“Does this scenario seem rational to you?”), by choosing “yes,” “sort of,” or “no.” They also specified how funny/rational the scenario was, from 0 (“not at all”) to 6 (“as funny/rational as possible”). Then, the participant reported the effort exerted for the task, from 0 (“none”) to 6 (“as much as possible”). Subsequently, the participants rated their emotions and subjective distress.

Next, during a delay period (about 20 min), the participants viewed a nature video with instructions to watch it carefully (15 min). They then reported how many times they thought about their stressful situation during the film (intrusive thoughts evaluation) and completed a multiple-choice knowledge test (subsequent performance evaluation) with eight questions about the video content (about 5 min). The video showed birds living in the forest and was assessed as neutral in the pilot study. Finally, the last measurement of emotions and subjective distress was taken. The experiment lasted 45–60 min. After the study, there was a debriefing session with a clinician, who explained the purpose of the study and attempted to identify and minimize any harm to the participants (no patient reported being harmed).

### Measurements

#### Diagnostic interview

The Structured Clinical Interview (SCID I)^57^ is a diagnostic interview administered by a trained interviewer to identify major mental disorders. The SCID I has exceptional inter-rater reliability and is considered an essential diagnostic tool in clinical research^64^.

#### Depressive symptoms

The Beck Depression Inventory-II (BDI-II)^47^ is a self-report scale containing 21 questions which measure depressive symptoms experienced over the past two weeks. In this study, Cronbach’s alpha was 0.57.

#### Attention efficiency

The d2 Test of Attention^49^ is one of the most widely used neuropsychological tests of selective attention. It is a cancellation test, which means it involves crossing out as many target stimuli as possible among similar non-target stimuli. The items are presented in 14 lines with 47 items in each line. The participant is permitted 20 s per line. The d2 Test has been proven to be an internally consistent and valid measure of attention^65^.

The study was conducted in accordance with the principles established in the 1975 Declaration of Helsinki, as revised in 2008, and was approved by the local ethics committee (The USSH Ethics Committee on Ethics of Empirical Research Involving People as Research Subjects; No 1/2015; G:2014/15/D/HS6/04991). The entire research was performed in accordance with relevant guidelines and regulations. Informed consent was obtained from all participants.

## Supplementary Information

Supplementary Information 1.

Supplementary Information 2.

## Data Availability

The dataset generated during and analysed during the current study is available in the *Mendeley Data* repository (http://dx.doi.org/10.17632/9rc79r8nyw.1).
