# Supplementary material for: Differential effects of stress-related and stress-unrelated humor in remitted depression
Source: Sci Rep. 2022 May 13;12:7946. doi: 10.1038/s41598-022-11515-y (PMC9106730; doi:10.1038/s41598-022-11515-y)
Supplement: Supplementary file 1 — Supplementary Information 1. [file 41598_2022_11515_MOESM1_ESM.rtf]

Abbreviations:
HRD    – humour related to distress
HURD – humour unrelated to distress
RER    – rational emotion regulation (control condition)

	Frequency (%)		
		
Unmedicated	
30 (31.9)	÷2(1) = 12.30 p < 0.001	
		Medicated	64 (68.1)		
		Antidepressants			
		Bupropion 	 2 (2.1)		
		Citalopram 	 1 (1.1)		
		Duloxetine 	 4 (4.3)		
		Escitalopram 	 13 (13.8)		
		Fluoxetine 	 2 (2.1)		
		Mirtazapine 	 2 (2.1)		
		Paroxetine 	 4 (4.3)		
		Sertraline 	 10 (10.6)		
		Trazodone 	 12 (12.8)		
		Venlafaxine	 19 (20.2)		
		Vortioxetine	 1 (1.1)		
		Antipsychotics			
		Chlorproxithene  	 5 (5.3)		
		Olanzapine 	 5 (5.3)		
		Quetiapine

Mood stabilizers
Lamotrigine
Lithium
Valproic Acid

Anxiolytics	
Buspirone	              
Hydroxyzine	
Pregabalin  
	 6 (6.4)


                2 (2.1)
                2 (2.1)
                9 (9.6)


                1 (1.1)
                2 (2.1)
                7 (7.4)	

	
