# Supplementary material for: Differential effects of stress-related and stress-unrelated humor in remitted depression
Source: Sci Rep. 2022 May 13;12:7946. doi: 10.1038/s41598-022-11515-y (PMC9106730; doi:10.1038/s41598-022-11515-y)
Supplement: Supplementary file 2 — Supplementary Information 2. [file 41598_2022_11515_MOESM2_ESM.rtf]

CONDITION 1: STRESS-RELATED HUMOR                (female version)
Creativity Task:
Transform Your Stressful Issue into Humor

Transform your fears related to the selected stressful issue into humorous fantasies                    by following these seven steps:

1) Start by admitting again what you fear could happen:
………………………………………………………………………………………………………………………………
2) Next ask, “And then what?” 
………………………………………………………………………………………………………………………………
3) Take your worry to the extreme by responding with an even worse outcome, immediately followed by, “And then what?”
………………………………………………………………………………………………………………………………
“And then what?”:
………………………………………………………………………………………………………………………………
4) Continue this process, exaggerating increasingly negative, even preposterous outcomes.
“And then what?”:
………………………………………………………………………………………………………………………………
“And then what?”:
………………………………………………………………………………………………………………………………
Continue for as long as you wish.

“And then what?”:
………………………………………………………………………………………………………………………………
“And then what?”:
………………………………………………………………………………………………………………………………
“And then what?”:
………………………………………………………………………………………………………………………………
“And then what?”:
………………………………………………………………………………………………………………………………
5) Finish by stating a final positive outcome

“And then what?”
………………………………………………………………………………………………………………………………

For example, you could write something similar to this:
I'm afraid that my company might downsize and I could lose my job.
“And then what?” … 
I would receive unemployment benefits while I look for another job.
“And then what?” … 
I won't find a job and my benefits will run out.
“And then what?” … 
I won't be able to pay my mortgage and will get evicted.
“And then what?” … 
I will have to ask my parents if I can move back home?
“And then what?” … 
They will say no and I will have to live in my car.
“And then what?” … 
I'll have no gas money and my car will get towed away with me inside.
“And then what?” … 
The towing company needs an office manager and they hire me!
6) Now, resume the question-and-answer session starting where you left off. 
“And then what?”
………………………………………………………………………………………………………………………………

7) This time, respond with exaggerated positive outcomes, each one more outrageous than the previous one. Continue until you feel safe or happy or proclaim your wildest dream.
For example:
The towing company needs an office manager and they hire me!
“And then what?” …                                                                                                                A client is impressed by my work and offers me a job in a corporate office!
“And then what?” …                                                                                                                                  My salary is tripled and I'm accepted into a management training program!
“And then what?” …                                                                                                                        I'm promoted to CEO and earn a six-figure income!

“And then what?”
………………………………………………………………………………………………………………………………
“And then what?”
………………………………………………………………………………………………………………………………
“And then what?”
………………………………………………………………………………………………………………………………


Continue for as long as you wish. 
Set no limitations on yourself. You can be whoever you wish and do whatever you desire.

“And then what?”:
………………………………………………………………………………………………………………………………
“And then what?”:
………………………………………………………………………………………………………………………………
“And then what?”:
………………………………………………………………………………………………………………………………
“And then what?”:
………………………………………………………………………………………………………………………………
“And then what?”:
………………………………………………………………………………………………………………………………
“And then what?”:
………………………………………………………………………………………………………………………………


Thank you!


CONDITION 2: STRESS-UNRELATED HUMOR      (female version)
Creativity Task:
Transform Someone's Stressful Issue into Humor

In a moment, you will be asked to create a humorous sequence of events regarding John's situation. Here is its description:
John, an IT specialist, is having a wedding next week. The day before, he had an accident and his legs were put in a cast. He worries about what will happen to the wedding ceremony.


Now, imagine John's stressful situation and transform it into comical fantasies 
by following these seven steps:

1) Start by writing what John fears could happen:
………………………………………………………………………………………………………………………………
2) Next ask, “And then what?” 
………………………………………………………………………………………………………………………………
3) Take his worry to the extreme by responding with an even worse outcome, immediately following with, “And then what?”
………………………………………………………………………………………………………………………………

“And then what?”:
………………………………………………………………………………………………………………………………


4) Continue this process, exaggerating increasingly negative, even preposterous outcomes.
“And then what?”:
………………………………………………………………………………………………………………………………
“And then what?”:
………………………………………………………………………………………………………………………………
Continue for as long as you wish.
“And then what?”:
………………………………………………………………………………………………………………………………
“And then what?”:
………………………………………………………………………………………………………………………………
“And then what?”:
………………………………………………………………………………………………………………………………
“And then what?”
………………………………………………………………………………………………………………………………
5) Finish by stating a final positive outcome.
“And then what?”
………………………………………………………………………………………………………………………………
An example:
Situation: Henry, an auto mechanic, decided to try working abroad. Upon his arrival,         he was waiting for hours for people arranging the job, and their phone was turned off. Henry worries he has been cheated...
For example, you could write something similar to this:
Henry was cheated. No one showed up, the number to call was off.
“And then what?” … 
He started looking for another job.
“And then what?” … 
He did not find a job and his money ran out.
“And then what?” … 
Henry asked his wife if he could come home with nothing.
“And then what?” … 
She said no. 
“And then what?” … 

Henry had to ask his wife if she would transfer him some money.
“And then what?” … 
She said no, so he had to live in his car and eat very little.
“And then what?” … 
Henry lost weight, nothing left of his tummy or his double chin. 
“And then what?” … 
A nearby nail salon needed a receptionist and they hired Henry!

6) Now, resume the question-and-answer session starting where you left off. 
“And then what?”
………………………………………………………………………………………………………………………………

7) This time, respond with exaggerated positive outcomes, each one more outrageous  than the previous one. Continue until you feel safe or happy or proclaim your wildest dream.
For example:
A nearby nail salon needed a receptionist and they hired Henry!
“And then what?” …                                                                                                                A client was impressed by his ideal figure and offered him to pose for swimsuit photos!
“And then what?” …                                                                                                                                  His salary was tripled and he was accepted into a prestigious model agency!
“And then what?” …                                                                                                                        Henry became a top model and started earning a six-figure income!

“And then what?”
………………………………………………………………………………………………………………………………
“And then what?”
………………………………………………………………………………………………………………………………
“And then what?”
………………………………………………………………………………………………………………………………

Continue for as long as you wish. 
Set no limitations on John. You can create whatever you wish.
“And then what?”:
………………………………………………………………………………………………………………………………
“And then what?”:
………………………………………………………………………………………………………………………………
“And then what?”:
………………………………………………………………………………………………………………………………


“And then what?”:
………………………………………………………………………………………………………………………………
“And then what?”:
………………………………………………………………………………………………………………………………


Thank you!


CONDITION 3: NON-HUMOROUS SCENARIO             (female version)
Creativity Task:
Transform Your Stressful Issue into a Scenario

Transform your fears related to the chosen stressful issue into a rational, plausible story by following these seven steps:

1) Start by admitting again what you fear could happen:
………………………………………………………………………………………………………………………………
2) If your worry does come true, what is most likely to happen?
………………………………………………………………………………………………………………………………
3) Next ask, “And then what?” 
………………………………………………………………………………………………………………………………
4) Continue this process, writing down further negative outcomes that are as realistic as possible.
“And then what?”:
………………………………………………………………………………………………………………………………
“And then what?”:
………………………………………………………………………………………………………………………………
Continue for as long as you wish.
“And then what?”:
………………………………………………………………………………………………………………………………
“And then what?”:
………………………………………………………………………………………………………………………………
“And then what?”:
………………………………………………………………………………………………………………………………
5) When you determine realistically what is the worst that could happen, write down a final positive outcome.
“And then what?”
………………………………………………………………………………………………………………………………


For example, you could write something similar to this:
I'm afraid that my company might downsize and I could lose my job.
“And then what?” … 
I would receive unemployment benefits while I look for another job.
“And then what?” … 
I won't find a good job.
“And then what?” … 
I will get some inferior job, for little pay.
“And then what?” … 
I will not be happy with this job. I will keep looking for a new one but to no avail.
“And then what?” … 
I will have to complete additional training to get a better job.
“And then what?” … 
I will spend a lot of time and money on this training.
“And then what?” … 
Finally, I got an interesting and well-paying job!
6) Now, resume the question-and-answer session starting where you left off. 
“And then what?”
………………………………………………………………………………………………………………………………

7) This time, respond with positive outcomes, each one slightly more optimistic than the previous one, but still rational and plausible. Continue until you feel safe or happy.
For example:
Finally, I got an interesting and well-paying job!
“And then what?” …                                                                                                                I started the job. I'm happy with it and doing well. 
“And then what?” …                                                                                                                                  My supervisor is impressed by my work and offers me a promotion.                                   “And then what?” …                                                                                                                        This job is the best one I have had so far. 

“And then what?”
………………………………………………………………………………………………………………………………
“And then what?”
………………………………………………………………………………………………………………………………
“And then what?”
………………………………………………………………………………………………………………………………


Continue for as long as you wish. 
Let your scenario be about positive, yet not exaggerated, events.

“And then what?”:
………………………………………………………………………………………………………………………………
“And then what?”:
………………………………………………………………………………………………………………………………
“And then what?”:
………………………………………………………………………………………………………………………………
“And then what?”:
………………………………………………………………………………………………………………………………
“And then what?”:
………………………………………………………………………………………………………………………………
“And then what?”:
………………………………………………………………………………………………………………………………


Thank you!
